# Predictive scoring models for persistent gram-negative bacteremia that reduce the need for follow-up blood cultures: a retrospective observational cohort study

**DOI:** 10.1186/s12879-020-05395-8

**Published:** 2020-09-17

**Authors:** Jongtak Jung, Kyoung-Ho Song, Kang Il. Jun, Chang Kyoung Kang, Nak-Hyun Kim, Pyoeng Gyun Choe, Wan Beom Park, Ji Hwan Bang, Eu Suk Kim, Sang-Won Park, Nam Joong Kim, Myoung-don Oh, Hong Bin Kim

**Affiliations:** 1 Department of Internal Medicine, Seoul National University Bundang Hospital, 82, Gumi-ro173 Beon-gil, Bundang-gu, Seongnam-si, Gyeonggi-do 13620 Republic of Korea; 2 Department of Internal Medicine, Seoul National University College of Medicine, Seoul, Republic of Korea

**Keywords:** Gram-negative bacteremia, Follow-up blood culture, Persistent bacteremia, Risk factor, Predictive scoring model

## Abstract

**Background:**

Although the risk factors for positive follow-up blood cultures (FUBCs) in gram-negative bacteremia (GNB) have not been investigated extensively, FUBC has been routinely carried out in many acute care hospitals. We attempted to identify the risk factors and develop a predictive scoring model for positive FUBC in GNB cases.

**Methods:**

All adults with GNB in a tertiary care hospital were retrospectively identified during a 2-year period, and GNB cases were assigned to eradicable and non-eradicable groups based on whether removal of the source of infection was possible. We performed multivariate logistic analyses to identify risk factors for positive FUBC and built predictive scoring models accordingly.

**Results:**

Out of 1473 GNB cases, FUBCs were carried out in 1268 cases, and the results were positive in 122 cases. In case of eradicable source of infection, we assigned points according to the coefficients from the multivariate logistic regression analysis: Extended spectrum beta-lactamase-producing microorganism (+ 1 point), catheter-related bloodstream infection (+ 1), unfavorable treatment response (+ 1), quick sequential organ failure assessment score of 2 points or more (+ 1), administration of effective antibiotics (− 1), and adequate source control (− 2). In case of non-eradicable source of infection, the assigned points were end-stage renal disease on hemodialysis (+ 1), unfavorable treatment response (+ 1), and the administration of effective antibiotics (− 2). The areas under the curves were 0.861 (95% confidence interval [95CI] 0.806–0.916) and 0.792 (95CI, 0.724–0.861), respectively. When we applied a cut-off of 0, the specificities and negative predictive values (NPVs) in the eradicable and non-eradicable sources of infection groups were 95.6/92.6% and 95.5/95.0%, respectively.

**Conclusions:**

FUBC is commonly carried out in GNB cases, but the rate of positive results is less than 10%. In our simple predictive scoring model, zero scores—which were easily achieved following the administration of effective antibiotics and/or adequate source control in both groups—had high NPVs. We expect that the model reported herein will reduce the necessity for FUBCs in GNB cases.

## Background

Although the positive rate of detection from follow-up blood cultures (FUBCs) in gram-negative bacteremia (GNB) is relatively low (5.8–10.9%) [1–3], and the risk factors for persistent GNB have not been investigated extensively, FUBCs have been routinely conducted in cases of GNB in many acute care hospitals [1–4]. Unnecessary routine blood cultures are invasive, and false positives due to contamination increase medical costs and time spent in hospitals [2, 5, 6]. The authors of a previous study identified the risk factors for persistent bacteremia, and fever was found to be the only risk factor associated with GNB [1]. Owing to the rarity of persistent GNB, the previous study had limitations, including an insufficient number of persistent GNB cases.

Recently, several studies have reported that a shorter course of antibiotics in uncomplicated GNB (hemodynamically stable patients who have received effective antibiotics and adequate source control) [7, 8] does not produce an inferior prognosis compared to a longer course. Further, a recent randomized control study showed that a 7-day course of antibiotic therapy in uncomplicated GNB was not inferior to a 14-day course. Thus, FUBCs may not be necessary for the management of uncomplicated GNB, since it can be adequately treated by a short course of antibiotics [8].

Therefore, we attempted to identify the risk factors for a positive FUBC result in GNB and developed a predictive scoring model to reduce the need for performing unnecessary FUBCs.

## Methods

### Patients

**Fig. 1:**
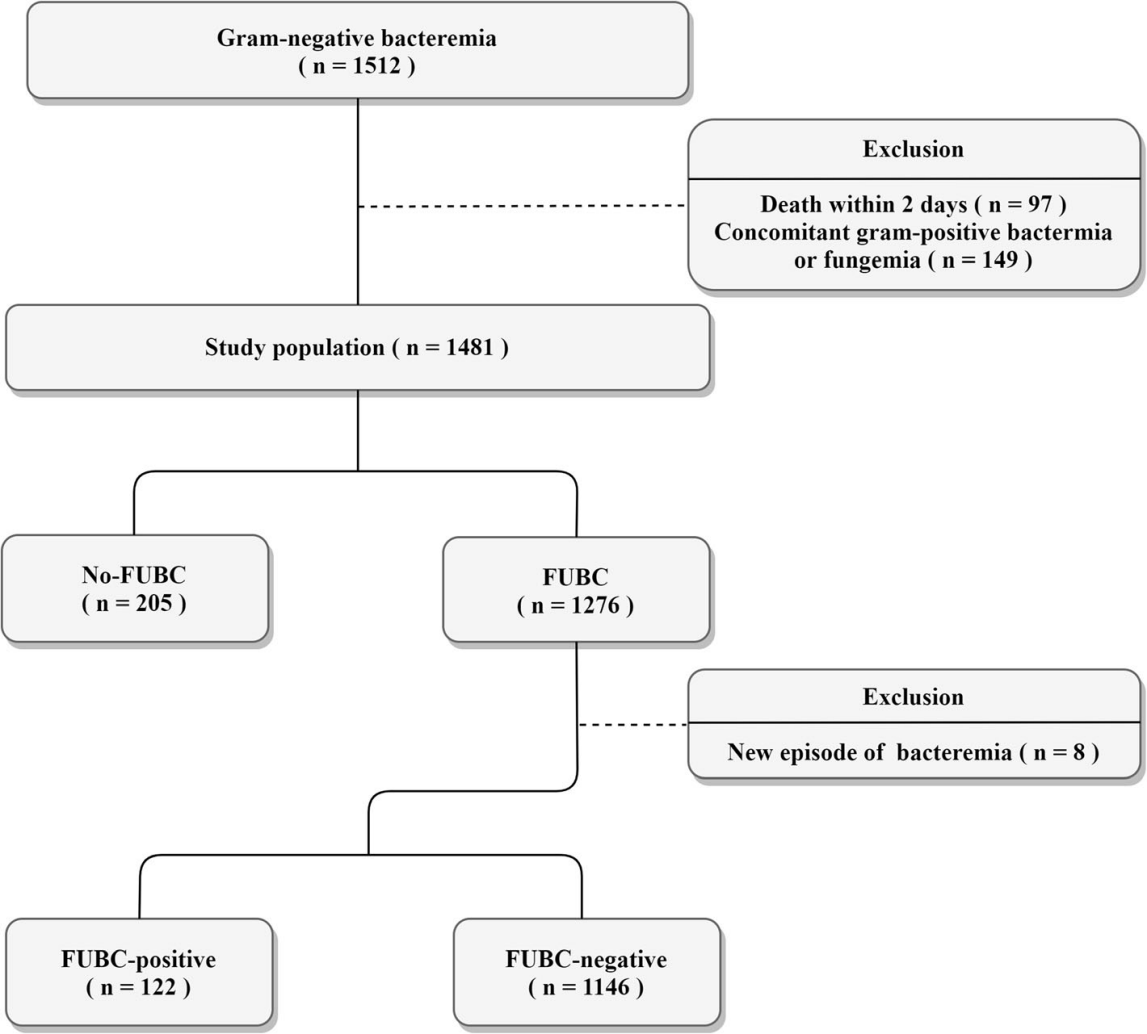
Flow chart of the study. Inclusion and exclusion criteria for the study population. FUBC: follow-up blood culture

We retrospectively reviewed all gram-negative episodes of bacteremia in a tertiary care university-affiliated 1300-bed hospital in South Korea from December 1, 2015 to December 1, 2017. Patients under 18 years of age, those who died within 2 days, and patients with concomitant gram-positive bacteremia or fungemia were excluded from the study (Fig. 1). New episodes of bacteremia identified by FUBC (different species were identified by the FUBC than those identified in the initial blood culture) were also excluded when we compared the FUBC-positive and -negative groups. The study was approved by the institutional review board of Seoul National University Bundang Hospital.

### Variables and groups

The variables were as follows: comorbidities, primary sources of infection, antibiotic status at the time of FUBC, identified microorganisms, susceptibility to antibiotics, fever, complete blood count, levels of C-reactive protein (CRP), quick sequential organ failure assessment (qSOFA) score, sequential organ failure assessment (SOFA) score, inotropic requirement, and source control status.

Patients who were subjected to FUBC 2–7 days after the initial blood culture, were assigned to the FUBC group, whereas patients who were not subjected to FUBC were assigned to the No-FUBC group. Cases were assigned to the FUBC-positive group if the same species of microorganism as the initial bacteremia was identified in at least one pair of FUBCs, and it was defined as persistent bacteremia. Cases in which bacteremia had been eliminated were assigned to the FUBC-negative group.

GNB was classified into eradicable and non-eradicable sources of infection according to the possibility of removal of the primary source of infection [9–11]; for example, removal of a central venous catheter or other endovascular devices; drainage of a dilated bile duct or hydronephrosis; surgical debridement of the skin, soft tissue infection, or osteomyelitis; drainage or removal of an intra-abdominal abscess; and drainage of empyema or a lung abscess.

An unfavorable treatment response was defined as the presence of at least two of the following variables: fever, aggravated leukocytosis, and no decrease in the level of CRP on the day that the FUBC was performed. Susceptible antibiotics were deemed to have been administered effectively when they were administered at least 1 calendar day before the FUBC according to the results of the antibiotics sensitivity test conducted in compliance with the Clinical and Laboratory Standards Institute (CLSI) guidelines. Adequate source control was defined as control of an eradicable source of infection at least 1 calendar day before the FUBC was performed [3, 10].

We compared the differences in clinical characteristics between the FUBC and the no-FUBC groups to assess selection bias. Subsequently, we divided the FUBC group into eradicable and non-eradicable sources of infection and performed multivariate logistic regression analyses to identify the independent risk factors for a positive FUBC. If species different from the one causing the initial bacteremia were identified in the FUBC, it was considered a new bacteremia and excluded from the analysis. We built a predictive scoring model by assigning scores according to the beta-coefficient of the logistic regression analyses and verified it using receiver operating characteristic curve (ROC) analysis.

### Statistical analyses

The statistical analyses were performed using IBM SPSS version 25.0. Fisher’s exact and Chi-square tests were used to analyze categorical variables, and student’s t-tests were used for continuous variables. Multivariate logistic regression analyses were performed using variables with *p*-values of less than 0.1 in the univariate analyses. A *p*-value of less than 0.05 was considered statistically significant in the multivariate analyses.

## Results

Overall, 1481 GNB cases were identified during the study period. Of these, FUBCs were performed in 1276 cases (86.2%), and 122 produced positive results (9.6%) (Fig. 1). There were no cases of suspected contamination, and a new bacteremia was identified in eight cases of FUBC (Supplementary Table 1). Comparisons between the FUBC group and the no-FUBC group are shown in Table 1. Variables such as hematologic malignancy, the presence of an intravascular device, and the presence of extended spectrum beta lactamase (ESBL)-producing microorganisms were significantly higher in the FUBC group. There was no significant difference in the incidence of in-hospital mortality between the two groups.

**Table 1:** Comparisons of clinical characteristics of gram-negative bacteraemia between the follow-up blood culture (FUBC) and no-FUBC groups

	FUBC-drawn (***n*** = 1276)	No FUBC-drawn (***n*** = 205)	***p***-value
**Age, years (± SD)**	68.85 (± 14.27)	69.2 (± 14.13)	0.744
**Sex (M)**	628 (49.2%)	117 (57.1%)	0.042*
**Body weight (kg)**	59.90 (± 11.56)	59.24 (± 12.62)	0.533
**Comorbidity**			
DM	404 (31.7%)	70 (34.1%)	0.519
Liver cirrhosis	95 (7.4%)	17 (8.3%)	0.776
ESRD on HD	2 (1.0%)	35 (2.7%)	0.154
ESRD on PD	6 (0.5%)	0 (0.0%)	1.000
Rheumatic disease	18 (1.4%)	1 (0.5%)	0.500
Haematologic malignancy	136 (10.7%)	7 (3.4%)	0.001*
Solid malignancy	361 (28.4%)	60 (29.4%)	0.802
Solid organ transplantation	34 (2.7%)	5 (2.4%)	1.000
Bone marrow transplantation	28 (2.2%)	1 (0.5%)	0.167
Intravascular device	308 (24.1%)	19 (9.3%)	0.000*
Neutropenia	127 (10.0%)	6 (2.9%)	0.002*
High-dose steroid	24 (1.9%)	1 (0.5%)	0.239
**Microorganism**			
*Escherichia coli*	722 (56.6%)	113 (55.1%)	0.705
*Klebsiella pneumoniae*	250 (19.6%)	45 (22.0%)	0.451
*Pseudomonas aeruginosa*	62 (4.9%)	7 (3.4%)	0.384
AmpC-encoded *Enterobacteriaceae*^a^	100 (7.8%)	21 (10.2%)	0.237
*Acinetobacter baumannii*	24 (1.9%)	1 (0.5%)	0.239
ESBL-producing	313 (24.5%)	32 (15.6%)	0.006*
Other gram-negative	110 (8.6%)	17 (8.3%)	0.895
PBSI	50 (3.9%)	8 (3.9%)	1.000
**Hospital onset**	328 (25.7%)	53 (25.9%)	1.000
**Site of infection**			
Urinary genital tract	502 (39.3%)	71 (34.6%)	0.217
Liver abscess	56 (4.4%)	7 (3.4%)	0.583
Biliary	303 (23.7%)	86 (42.0%)	0.000*
Intra-abdominal	135 (10.6%)	18 (8.8%)	0.462
Respiratory	56 (4.4%)	5 (2.4%)	0.255
SSTI	13 (1.0%)	1 (0.5%)	0.707
Catheter-related	40 (3.1%)	2 (1.0%)	0.109
Bone and joint infection	12 (0.9%)	1 (0.5%)	1.000
Cardiovascular	3 (0.2%)	0 (0.0%)	1.000
CNS infection	3 (0.2%)	0 (0.0%)	1.000
Primary bacteraemia	143 (11.2%)	10 (4.9%)	0.006*
**In-hospital mortality**	87 (6.8%)	18 (8.8%)	0.379

*SD* Standard deviation, *M* Male, *DM* Diabetes mellitus, *ESRD* End-stage renal disease, *HD* Haemodialysis, *PD* Peritoneal dialysis, *ESBL* Extended-spectrum beta-lactamase, *PBSI* Polymicrobial bloodstream infection, *SSTI* Skin and soft tissue infection, *CNS* Central nervous system

^a^AmpC-encoded *Enterobacteriaceae* includes *Serratia marcescens*, *Providencia stuartii*, *Proteus vulgaris*, *Citrobacter spp.*, *Enterobacter spp.*, and *Morganella morganii*

*p < 0.05

**Fig. 2:**
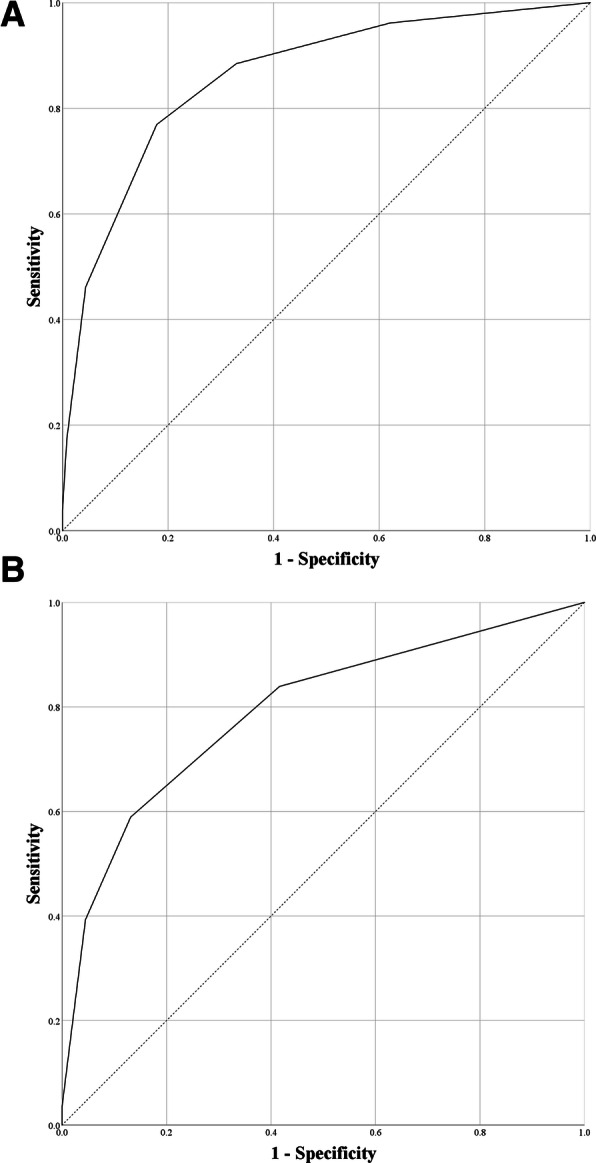
**a** Receiver operating characteristic (ROC) analysis of the predictive scoring model in cases with eradicable sources of infection. The area under the curve (AUC) was 0.861 (95% confidence interval (CI) 0.806–0.916). **b** ROC analysis of the predictive scoring model in cases with non-eradicable sources of infection. The AUC was 0.792 (95% CI, 0.724–0.861)

Positive and negative clinical characteristics in the FUBC groups are compared in Table 2, according to the eradicability of the infection source, and the identified independent risk factors are listed in Table 3. In cases where the source of infection was eradicable, the independent risk factors were as follows: the presence of ESBL-producing microorganisms, catheter-related bloodstream infection, unfavorable treatment responses, a qSOFA score of at least 2 points, the administration of effective antibiotics, and adequate source control. In cases where the source of infection was non-eradicable, the independent risk factors were as follows: end-stage renal disease on hemodialysis, unfavorable treatment responses, and the administration of effective antibiotics. We assigned points to the independent risk factors for positive FUBC, based on the beta-coefficients from the logistic regression analysis (Table 3). In cases of eradicable and non-eradicable sources of infection, the values of the area under the curve of the receiver operating characteristic curve (AUC-ROC) of each scoring model were 0.861 (95% confidence interval (CI) 0.806–0.916) and 0.792 (95% CI, 0.724–0.861), respectively (Table 3, Fig. 2). The sensitivities, specificities, positive predictive values (PPVs), and negative predictive values (NPVs) according to the cut-off values are listed in Table 4.

**Table 2:** Clinical characteristics of gram-negative bacteraemia according to the results of follow-up blood culture (FUBC) and the eradicability of the source of infection

	Eradicable source of infection			Non-eradicable source of infection		
	Positive FUBC (*n* = 55)	Negative FUBC (*n* = 411)	*p*-value	Positive FUBC (*n* = 66)	Negative FUBC (*n* = 736)	*p*-value
**Age, years (± SD)**	69.18 (± 12.19)	70.15 (± 13.35)	0.610	70.15 (± 14.25)	67.98 (± 14.88)	0.254
**Sex (M)**	29 (52.7%)	222 (54.0%)	0.886	26 (39.4%)	346 (47.0%)	0.235
**Body weight (kg)**						
**Comorbidity**						
DM	19 (34.5%)	121 (29.4%)	0.531	25 (37.9%)	238 (32.3%)	0.358
Liver cirrhosis	6 (10.9%)	31 (7.5%)	0.422	4 (6.1%)	54 (7.3%)	1.000
ESRD on HD	4 (7.3%)	7 (1.7%)	0.031*	6 (9.1%)	18 (2.4%)	0.010*
ESRD on PD	0 (0.0%)	0 (0.0%)	N.A.	2 (3.0%)	4 (.0.5%)	0.081
Rheumatic disease	1 (1.8%)	0 (0.0%)	0.119	2 (3.0%)	15 (2.0%)	0.644
Haematologic malignancy	4 (7.3%)	8 (2.0%)	0.042*	12 (18.2%)	109 (14.9%)	0.474
Solid malignancy	17 (30.9%)	149 (36.4%)	0.457	16 (24.2%)	176 (24.0%)	0.966
Solid organ transplantation	2 (3.6%)	9 (2.2%)	0.627	4 (6.1%)	19 (2.6%)	0.113
Bone marrow transplantation	2 (3.6%)	1 (0.2%)	0.038*	2 (3.0%)	22 (3.0%)	1.000
Intravascular device	75 (18.2%)	26 (47.3%)	0.000*	4 (6.1%)	19 (2.6%)	0.113
Neutropenia	1 (1.8%)	8 (2.0%)	1.000	11 (16.7%)	105 (14.3%)	0.608
High-dose steroid	1 (1.8%)	4 (1.0%)	0.468	4 (6.1%)	15 (2.0%)	0.063
**Microorganism**						
*Escherichia coli*	22 (40.0%)	205 (49.9%)	0.197	36 (54.5%)	456 (62.0%)	0.291
*Klebsiella pneumoniae*	11 (20.0%)	91 (22.1%)	0.735	14 (21.2%)	133 (18.1%)	0.527
*Pseudomonas aeruginosa*	5 (9.1%)	19 (4.6%)	0.185	5 (7.6%)	33 (4.5%)	0.231
vAmpC-encoded *Enterobacteriaceae*^a^	7 (12.7%)	49 (11.9%)	1.000	4 (6.1%)	39 (5.3%)	0.774
*Acinetobacter baumannii*	1 (1.8%)	6 (1.5%)	0.587	2 (3.0%)	15 (2.0%)	0.644
ESBL-producing	21 (38.2%)	84 (20.4%)	0.003*	39 (59.1%)	169 (23.0%)	0.000*
Other gram-negative	9 (16.4%)	34 (8.3%)	0.052*	5 (7.6%)	59 (8.0%)	0.899
PBSI	5 (9.1%)	32 (7.8%)	0.789	1 (1.5%)	12 (1.6%)	1.000
**Hospital onset**	22 (40.0%)	66 (16.1%)	0.000*	19 (28.8%)	216 (29.3%)	1.000
**Site of infection**						
Urinary genital tract	12 (21.8%)	80 (19.5%)	0.718	38 (57.6%)	372 (50.5%)	0.305
Liver abscess	2 (3.6%)	39 (9.5%)	0.205	0 (0.0%)	13 (1.8%)	0.615
Biliary infection	9 (16.4%)	218 (53.0%)	0.000*	1 (1.5%)	73 (9.9%)	0.024*
Intra-abdominal	5 (9.1%)	38 (9.2%)	1.000	6 (9.1%)	85 (11.5%)	0.687
Respiratory	1 (1.8%)	2 (0.5%)	0.315	7 (10.6%)	44 (6.0%)	0.180
SSTI	3 (5.5%)	4 (1.0%)	0.039*	2 (3.0%)	4 (0.5%)	0.081
Catheter-related	18 (32.1%)	22 (5.4%)	0.000*	0 (0.0%)	0 (0.0%)	N.A.
Bone and joint infection	3 (5.5%)	2 (0.5%)	0.013*	1 (1.5%)	6 (0.8%)	0.453
Cardiovascular	2 (3.6%)	1 (0.2%)	0.038*	0 (0.0%)	0 (0.0%)	N.A.
CNS infection	0 (0.0%)	0 (0.0%)	N.A.	0 (0.0%)	2 (0.2%)	1.000
Primary bacteraemia	0 (0.0%)	0 (0.0%)	N.A.	11 (16.7%)	131 (17.8%)	0.869
**Inotropic requirement on the day of FUBC**	24 (5.8%)	10 (18.2%)	0.003*	9 (13.6%)	53 (7.2%)	0.061
**Unfavourable treatment response**	30 (55.6%)	130 (32.5%)	0.001*	31 (48.4%)	215 (30.0%)	0.002*
**qSOFA score (± SD) on the day of FUBC**	1.29 (± 1.12)	0.52 (± 0.76)	0.000*	0.88 (± 0.95)	0.56 (± 0.88)	0.005*
**SOFA score (± SD) on the day of FUBC**	5.62 (± 4.48)	3.09 (± 2.93)	0.000*	3.79 (± 3.47)	2.90 (± 3.18)	0.036*
**qSOFA score ≥ 2 on the day of FUBC**	20 (36.4%)	45 (10.9%)	0.000*	16 (24.2%)	108 (14.7%)	0.039
**Effective antibiotics before the day of FUBC**	34 (61.8%)	350 (85.2%)	0.000*	27 (40.9%)	639 (86.8%)	0.000*
**Adequate source control before the day of FUBC**	13 (23.6%)	322 (78.3%)	0.000*	N.A.	N.A.	N.A.

*SD* Standard deviation, *N.A*. Not available, *M* Male, *DM* Diabetes mellitus, *ESRD* End-stage renal disease, *HD* Haemodialysis, *PD* Peritoneal dialysis, *ESBL* Extended-spectrum beta-lactamase, *PBSI* Polymicrobial bloodstream infection, *SSTI* Skin and soft tissue infection, *CNS* Central nervous system

^a^AmpC-encoded *Enterobacteriaceae* includes *Serratia marcescens*, *Providencia stuartii*, *Proteus vulgaris*, *Citrobacter spp.*, *Enterobacter spp.*, and *Morganella morganii*

*p < 0.05

**Table 3:** Independent risk factors and assigned scores used to build the predictive scoring model for positive follow-up blood culture in gram-negative bacteraemia, according to eradicable and non-eradicable sources of infection

Eradicable source of infection				
	Beta-coefficient	Odds ratio (95% CI)	*p*-value	Assigned score
ESBL-producing microorganism infection	1.001	2.720 (1.179–6.271)	0.019	+ 1
CRBSI	1.374	3.95 (1.522–10.255)	0.005	+ 1
Unfavourable treatment response^a^	0.802	2.229 (1.262–3.937)	0.006	+ 1
qSOFA score ≥ 2 on the day of FUBC	0.864	2.371 (1.034–5.438)	0.041	+ 1
Effective antibiotics administration before the day of FUBC	−1.007	0.365 (0.164–0.814)	0.014	−1
Adequate source control before the day of FUBC	−1.983	0.138 (0.064–0.294)	0.000	−2
**Non-eradicable source of infection**				
ESRD on HD	1.406	4.081 (1.331–12.515)	0.014	+ 1
Unfavourable treatment response^a^	0.802	2.229 (1.262–3.937)	0.006	+ 1
Effective antibiotics administration before the day of FUBC	−2.015	0.133 (0.069–0.258)	0.000	−2

*CI* Confidence interval, *ESBL* Extended-spectrum beta-lactamase, *CRBSI* Catheter-related bloodstream infection, *qSOFA* Quick sequential organ failure assessment, *FUBC* Follow-up blood culture, *ESRD* End-stage renal disease, *HD* Haemodialysis

^a^Unfavourable treatment response was defined as positivity for at least 2 variables among the presence of fever, aggravated leucocytosis, and no decrease of C-reactive protein on the day of FUBC

**Table 4:** Receiver operating characteristics and predictability of scoring models for positive follow-up blood culture in gram-negative bacteraemia, according to the various cut-off values

	AUC (95% CI)	Cut-off	Sensitivity	Specificity	PPV	NPV
Eradicable source of infection^a^	0.861 (0.806–0.916)	−2	88.5%	67.0%	27.7%	97.6%
		−1	76.9%	82.1%	38.1%	96.1%
		0	46.2%	95.6%	60.0%	92.6%
		1	17.3%	99.3%	75%	90.63%
		2	3.8%	100%	100%	87.9%
Non-eradicable source of infection	0.792 (0.724–0.861)	−2	83.9%	58.4%	14.4%	97.6%
		−1	58.9%	86.9%	27.3%	96.2%
		0	39.3%	95.5%	42.3%	95.0%
		1	3.6%	100.0%	100.0%	92.5%

*AUC* Area under the curve, *CI* Confidence interval, *PPV* Positive predictive value, *NPV* Negative predictive value

^a^Gram-negative bacteraemia in which the primary source of infection could be removed, e.g. removal of a central venous catheter or other endovascular device, drainage of a dilated bile duct or hydronephrosis, surgical debridement of skin and soft tissue infection or osteomyelitis, drainage or removal of an intra-abdominal abscess, or drainage of empyema or a lung abscess

When we applied a cut-off value of 0, the specificities of the eradicable and non-eradicable sources of infection were 95.6 and 95.5%, respectively. The NPVs of the predictive scoring models in the eradicable and non-eradicable source infections were 92.6 and 95.0%, respectively.

**Fig. 3:**
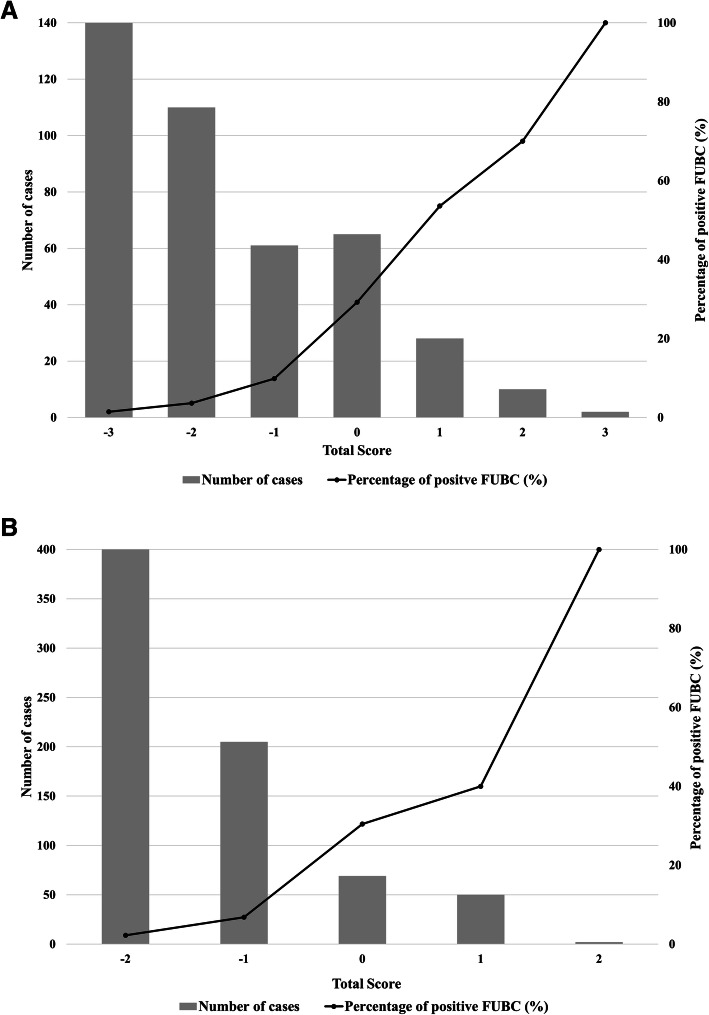
**a** The percentage of positive follow-up blood cultures according to the scores of eradicable and **b** non-eradicable source of infection

The percentage of positive FUBCs according to the scores of eradicable and non-eradicable source of infection is shown in Fig. 3. The percentage of positive FUBCs was less than 2% in cases with − 3 or − 2 points of eradicable or non-eradicable sources of infection, but 40 and 70% had positive FUBCs in cases with 1 or 2 points of non-eradicable or eradicable sources of infection, respectively.

## Discussion

Our study revealed that FUBCs were performed in most of the patients with GNB, but less than 10% produced positive results. In contrast to gram-positive bacteremia, positive FUBC results were not common in GNB [1, 3].

In eradicable sources of infection, variables such as ESBL-producing organisms, catheter-related bloodstream infections (CRBSIs), unfavorable treatment responses, and qSOFA scores of at least 2 were independent risk factors, but when effective antibiotics were administered and there was adequate source control the day before performing FUBC, the probability of negative conversion of bacteremia in FUBC increased significantly. When appropriate management (e.g., administration of effective antibiotics and source control) was performed, and there was a clear clinical response (qSOFA score < 2), the score predicted by the model did not exceed the cut-off value (zero points), and the probability of negative conversion in FUBC was as high as 92.6%. In *Staphylococcus aureus* infections, delays in the removal of eradicable sources of infection, the initial administration of inappropriate antibiotics, and delays in the delivery of appropriate antibiotics were important risk factors for persistent bacteremia [10, 12]. Early source control (within 48 h) was also important for eradicating bacteremia in both gram-positive and gram-negative bacteremia [3]. Therefore, as with *S. aureus* bacteremia, in cases of GNB with eradicable sources of infection—regardless of the site of infection, underlying diseases, or causative microorganisms—if there is appropriate management and the clinical response is favorable, FUBC provides little benefit.

In cases in which the source of infection was non-eradicable, hemodialysis and an unfavorable treatment response were independent risk factors. However, if effective antibiotics were administered to the patient, bacteremia was usually eliminated. If effective antibiotics were administered—regardless of the underlying disease, microorganism type, or treatment response—the score did not exceed the cut off-value (zero points), and the probability of negative conversion of bacteremia was 95.0%.

If FUBC was performed only in cases with more than zero points of both eradicable and non-eradicable source of infection, more than 90% of FUBCs could have been avoided in our study population (Fig. 3). Even if the cutoff value was more strictly set as − 1 to increase the NPV, more than 70% of FUBCs could have been saved. Therefore, performing FUBC to evaluate the response to treatment can be avoided in most uncomplicated cases of GNB. In GNB cases, FUBC could be applied selectively to patients with a high risk of positive FUBCs, unlike in *S. aureus* bacteremia or candidemia.

The contamination rate of peripheral blood cultures in our hospital is as low as 0.5%, and among the 1276 cases of FUBC in our study, there were no suspected cases of contamination. However, contamination rates in blood cultures have been reported ranging from 0.9–7.9% [5]. In other studies related to FUBCs, contaminants were identified in 2.0–3.9% of FUBCs [13, 14]. Therefore, universal FUBCs of all gram-negative bacteremia may produce contamination, which could lead to increased medical costs and prolonged hospital stays [5].

Recently, one study revealed that FUBC was a useful diagnostic tool for differentiating septic thrombophlebitis in gram-negative bacteremia of patients admitted to the intensive care unit (ICU) for polytrauma [15]. If the patient had a high-risk of persistent GNB and risk factors for deep vein thrombosis, such as the presence of a central catheter, multiple traumas or admission to the ICU [16, 17], further diagnostic evaluation, such as CT angiography and duplex sonography as well as FUBCs to differentiate septic thrombophlebitis, could be considered.

Recent studies revealed that when FUBCs were performed in cases of gram-negative bloodstream infection, they were associated with improved clinical outcomes [13, 14]. However, the proportion of FUBCs performed was relatively low (17.6–68%) compared to our present study (86.2%). In addition, the rates of positive FUBC (20–38.5%) and all-cause mortality (10–13.7%) were higher than in our study population (9.7% positive-FUBC rate and 6.8% in-hospital mortality in the FUBC group). This means that FUBCs were selectively performed in severe GNB cases in these previous studies. It cannot be concluded that FUBC improves the prognosis in all GNB, especially in uncomplicated bacteremia. The FUBC group in these studies had a trend of longer treatment duration and hospital stays than the no-FUBC group, and contaminants were identified in 2.0–3.9% of FUBCs. If FUBC is conducted routinely in uncomplicated GNB, it may increase medical costs and cause various side effects due to prolonged antibiotic treatment. Therefore, the decision of conducting an FUBC should be made carefully, and our predictive scoring model will help with that decision. Appropriately conducted FUBCs, according to the results of our predictive scoring model, may improve clinical outcomes and reduce side effects and medical costs.

Our study has some limitations. First, there may have been bias towards the FUBC group, because the study was conducted retrospectively. However, variables such as hematologic malignancy, presence of an intravascular device, and ESBL-producing microorganisms—which were significantly more prevalent in the FUBC group than in the no-FUBC group— were also significant risk factors for positive FUBCs (Table 1). This means that there was a higher probability of persistent bacteremia in the FUBC group than in the no-FUBC group. The NPVs of predictive scoring models in real-world cases of GNB would be higher than those indicated in the present study. Therefore, this selection bias did not alter our conclusion. Second, we did not determine how FUBC affects patient outcomes such as mortality, morbidity, length of stay in hospital, or total cost of medical care. Therefore, our findings alone will not be sufficient to change routine practice. As discussed above, recent studies have shown that FUBC was associated with improved outcome of GNB [13, 14], but these results couldn’t be applied to the uncomplicated GNB. Further investigations such as a prospective randomized control study should be conducted to reveal the exact clinical impact of FUBC in GNB.

## Conclusions

In this study, although FUBC was commonly used in cases of GNB, there were few positive results (< 10% of cases). We expect that the application of our simple predictive scoring model will reduce the need for performing unnecessary FUBC in uncomplicated cases of GNB.

## Supplementary information

**Additional file 1 Table S1**. Summarized 8 cases of new bacteremia.

## Data Availability

The datasets used and/or analyzed during the study are available from the corresponding author on reasonable request.

## Abbreviations

FUBC: Follow-up blood culture
GNB: Gram-negative bacteremia
CRP: C-reactive protein
qSOFA) score: Quick sequential organ failure assessment
ESBL: Extended spectrum beta lactamase
CI: Confidence interval
PPVs: Positive predictive values
NPVs: Negative predictive values
CRBSI: Catheter-related bloodstream infection
