# Supplementary material for: Predictive scoring models for persistent gram-negative bacteremia that reduce the need for follow-up blood cultures: a retrospective observational cohort study
Source: BMC Infect Dis. 2020 Sep 17;20:680. doi: 10.1186/s12879-020-05395-8 (PMC7499917; doi:10.1186/s12879-020-05395-8)
Supplement: Supplementary file 1 — Additional file 1 Table S1. Summarized 8 cases of new bacteremia. [file 12879_2020_5395_MOESM1_ESM.docx]

**SUPPLEMENTARY MATERIAL**

Supplementary Table 1. Summarized 8 cases of new bacteremia

| Comorbidity | Site of infection | Result of Initial blood culture | Result of FUBC |
| --- | --- | --- | --- |
| Hepatocellular carcinoma | Cholangitis, CRBSI | *Serratia marcescens* | *Stenotrophomonas maltophilia* |
| Cushing syndrome | Primary bacteremia, CRBSI | *Klebsiella pneumoniae* | *Staphylococcus aureus* |
| ALL | Pneumonia, Primary bacteremia | *Stenotrophomonas maltophilia* | *Enterococcus faecium* |
| Cholangiocarcinoma | Cholangitis | *Escherichia coli* | *Pseudomonas aeruginosa, Enterococcus casseliflavus* |
| Cholangiocarcinoma | Cholangitis | *Moraxella species* | *Pseudomonas aeruginosa* |
| HLH, AlloHCT | Primary bacteremia | *Escherichia coli* | *Pseudomonas aeruginosa* |
| Multiple myeloma | Cholangitis | *Escherichia coli* | *Citrobacter freundii* |
| BPF, chronic empyema | Pneumonia | *Providencia stuartii* | *Pseudomonas aeruginosa* |

FUBC, follow-up blood culture; CRBSI, catheter-related bloodstream infection; ALL, Acute lymphoblastic leukemia; HLH, hemophagocytic lymphohistiocytosis; AlloHCT, allogenic hematopoietic cell transplantation; BPF, Bronchopulmonary fistula
